# Immunotherapy against glioblastoma using backpack‐activated neutrophils

**DOI:** 10.1002/btm2.10712

**Published:** 2024-08-13

**Authors:** Tatsuya Fukuta, Ninad Kumbhojkar, Supriya Prakash, Suyog Shaha, A. Da Silva‐Candal, Kyung Soo Park, Samir Mitragotri

**Affiliations:** ^1^ Harvard John A. Paulson School of Engineering and Applied Sciences, Harvard University Boston Massachusetts USA; ^2^ Wyss Institute for Biologically Inspired Engineering Boston Massachusetts USA; ^3^ Present address: Department of Physical Pharmaceutics, School of Pharmaceutical Sciences, Wakayama Medical University, Japan Wakayama Japan; ^4^ Present address: Neurovascular Diseases Laboratory, Neurology Service, University Hospital Complex of A Coruña, Biomedical Research Institute A Coruña Spain

**Keywords:** cell therapy, glioblastoma, immune checkpoint inhibitor, immunotherapy, neutrophils

## Abstract

Immune checkpoint inhibitors (ICIs) represent new therapeutic candidates against glioblastoma multiforme (GBM); however, their efficacy is clinically limited due to both local and systemic immunosuppressive environments. Hence, therapeutic approaches that stimulate local and systemic immune environments can improve the efficacy of ICIs. Here, we report an adoptive cell therapy employing neutrophils (NE) that are activated via surface attachment of drug‐free disk‐shaped backpacks, termed Cyto‐Adhesive Micro‐Patches (CAMPs) for treating GBM. CAMP‐adhered neutrophils (NE/CAMPs) significantly improved the efficacy of an anti‐PD1 antibody (aPD‐1) in a subcutaneous murine GBM model (GL261). A combination of NE/CAMPs and aPD‐1 completely regressed subcutaneous GL261 tumors in mice. The efficacy of NE/CAMPs against GBM was also tested in an orthotopic GL261 model. Neutrophil's ability to migrate into the brain was not affected by CAMP attachment, and intracerebral NE/CAMP accumulation was observed in mice‐bearing orthotopic GBM. The combination treatment of NE/CAMPs and aPD‐1 activated systemic immune responses mediated by T cells and showed improved therapeutic responses compared with aPD‐1 alone in the orthotopic GBM model. These results suggest that immunomodulation with NE/CAMPs offers a potential approach for the treatment of GBM by combination with ICIs.

**Translational Impact Statement**

The manuscript describes a novel potential treatment for glioblastoma multiforme. The method utilizes neutrophils, the most abundant circulatory immune cells, activated by a drug‐free polymer micro‐patch. Activated neutrophils infiltrate into the tumor and alter the tumor microenvironment to improve their susceptibility to check‐point inhibitors. This method adds to the repertoire of techniques as potential treatments for glioblastoma multiforme, a cancer that is currently challenging to treat.

## 1 INTRODUCTION

Glioblastoma multiforme (GBM) is an aggressive, fatal, and the most malignant brain tumor, which accounts for over 50% of brain tumors.^1^ The current standard therapy in clinical settings is surgical resection with a maximal safe approach, followed by radiation therapy and pharmacotherapy such as temozolomide and bevacizumab.^2^ However, due to the limited therapeutic efficacy of these standard therapies, the median survival rate of GBM patients is only 15–17 months after definitive diagnosis, and the 5‐year survival remains less than 10%.^3^ Unfortunately, the clinical efficacy of current treatments has not meaningfully improved over 30 years^4^; thus GBM remains one of the most difficult cancers to treat.

Application of immunotherapies including immune checkpoint inhibitors (ICIs) and tumor antigen vaccines has been proposed to improve therapeutic outcomes against GBM.^5^ Among them, significant efforts have been carried out using ICIs due to their demonstrated clinical benefits in other intractable cancers including metastatic melanoma, non‐small cell lung cancer, and renal cell carcinoma.^6^ However, results obtained in other cancers have not been clinically translated to GBM and no survival benefits have been observed compared to the standard of care.^7^ In the first phase III study with anti‐programmed cell death‐1 (aPD‐1) termed Checkmate 143 (NCT02017717), patients with recurrent GBM received either aPD‐1 immunotherapy with nivolumab or anti‐vascular endothelial growth factor treatment with bevacizumab resulting in no improvement in overall survival.^8^ As a part of another phase III study Checkmate 548 (NCT02667587), patients with newly diagnosed GBM were treated with a combination of aPD‐1 nivolumab and current standard‐of‐care (radiotherapy and temozolomide) and the combination yielded no significant improvement over the standard of care alone.^9^ Some pre‐clinical studies have reported improved therapeutic response in GBM models with the combination of aPD‐1 and other immunotherapies including anti‐cytotoxic T‐lymphocyte antigen‐4 (aCTLA‐4).^10^, ^11^, ^12^ The primary reasons for the disappointing results with ICI monotherapy against GBM have been attributed to the presence of the blood–brain barrier (BBB), blood‐tumor barrier (BTB), and highly immunosuppressive tumor microenvironment (TME) composed of tumor‐associated macrophages and other immune‐related cells.^13^, ^14^ Monoclonal antibodies such as aPD‐1 tend to show poor penetration across the BBB due to their large molecular size. However, it is also reported that aPD‐1 irreversibly binds to PD‐1 on peripheral circulating T cells and can ultimately penetrate the BBB into the brain parenchyma,^15^, ^16^ although aPD‐1 monotherapy shows insufficient therapeutic response against GBM due to immunosuppressive TME as mentioned above. In addition to the local TME in the brain, systemic immunosuppression also contributes to the ineffectiveness of immunotherapies in GBM patients. Systemic immunosuppression is displayed in multiple ways such as reduced count and functionality of T cells,^17^ reduced major histocompatibility complex‐II levels in blood‐derived monocytes,^18^ sequestration of T cells in bone marrow,^19^ and destruction of antigen‐primed antigen‐presenting cells (APCs) as well as T cells in secondary lymphoid organs,^20^ resulting in inactivation of peripheral APCs and loss of adaptive immune function. Although the detailed mechanisms underlying systemic immunosuppression are not fully understood, those events related to systemic immunosuppression also lead to a decrease in the therapeutic efficacy of ICIs in GBM. Hence, improvement of local brain TME as well as systemic immunosuppression are required to induce a potent immune response against GBM and enhanced therapeutic outcomes of ICI treatment.

Adoptive cell therapy has been recently reported as a promising therapeutic option against GBM. For instance, the usefulness of genetically engineered T cells expressing chimeric antigen receptors (CARs) has been demonstrated in preclinical studies and several clinical trials that employ CAR‐T cells specifically targeting brain tumor‐associated antigens such as epidermal growth factor variant III (EGFRvIII), and interleukin‐13 receptor alpha 2 (IL‐13Rα2) are ongoing.^21^ In these studies, T cell exhaustion by the immunosuppressive environment has been shown to hamper therapeutic outcomes.^22^ Circulating immune cells (e.g., neutrophils and monocytes) have an intrinsic capability to pass through the BBB and infiltrate into brain tumor mass by responding to biological cues.^23^ Leveraging this attribute, some preclinical studies have demonstrated the use of neutrophils (NEs) as a delivery vehicle of anti‐cancer drug‐loaded nanoparticles to achieve their selective delivery to tumor tissue for treating glioma.^24^, ^25^ These studies employed NEs as a drug delivery tool rather than therapeutic cells. We have recently demonstrated the novel potential of the adoptive transfer of activated NEs for cancer immunotherapy.^26^ NE activation was achieved by surface attachment of disk‐shaped polylactic‐co‐glycolic acid (PLGA)‐based microparticulate backpacks with high aspect ratio, termed “Cyto‐Adhesive Micro‐Patches (CAMPs).” This phenomenon is primarily based on the observation that the physical adhesion of NEs to macroscopic surfaces induces their activation via frustrated phagocytosis.^27^, ^28^ Integrin‐mediated attachment of drug‐free CAMPs rapidly activated NEs without any biological stimulation or genetic modification, and increased expressions of pro‐inflammatory genes and proteins, resulting in the conversion of NE phenotype to the anti‐tumor N1 type.^26^

Moreover, the adoptive transfer of CAMP‐activated NEs (NE/CAMPs) induced robust anti‐tumor systemic immune response through activation of immune cells and suppressed tumor growth in mice burdened with breast cancer and melanoma tumors. The treatment with NE/CAMPs further exhibited a synergistically improved therapeutic efficacy when combined with immune checkpoint inhibitors targeting CTLA‐4 and PD‐1.^26^ Although detailed roles of NEs in GBM are currently unclear, NEs exhibit both anti‐tumor N1 and pro‐tumor N2 phenotypes under GBM conditions, similar to the TME in other cancers, and N1‐type NEs have been reported to contribute to tumor growth suppression, especially in the early stage of GBM development.^29^, ^30^ Based on these findings, we hypothesized that activation of systemic anti‐tumor immune response by CAMP‐activated NE treatment could improve immunosuppressive environment, which concomitantly would synergize with ICI such as aPD‐1, resulting in a positive therapeutic outcome in case of GBM.

Here, we investigated the therapeutic efficacy of NE/CAMPs for the treatment of GBM in GL261 tumor‐bearing mice. The surface of CAMPs was modified with the Fab fragment of anti‐CD11b antibody to promote their attachment via CD11b expressed abundantly on NE membranes. By using the subcutaneous GL261 model, we first compared the therapeutic efficacy of NE/CAMPs with NE alone and also evaluated the effect of their combined treatment with aPD‐1. aPD‐1 was used in this study since the large number of ongoing clinical trials using aPD‐1 for GBM therapy, some of which have reached phase III.^31^, ^32^ We next evaluated the efficacy of the combination of NE/CAMPs and aPD‐1 treatments in the orthotopic GL261 tumor‐bearing mice. Finally, we assessed the influence of CAMP attachment on the ability of NEs to migrate into the GBM tissue as well as the effect of NE/CAMP+aPD‐1 treatment on the immune responses in orthotopic GL261 tumors. The results demonstrate, for the first time, the therapeutic potential of NE‐based adoptive cell therapy for GBM, one of the most difficult tumors to treat.

## 2 RESULTS

### 2.1 Polymeric CAMPs can be efficiently attached to the surface of neutrophils

**FIGURE 1:**
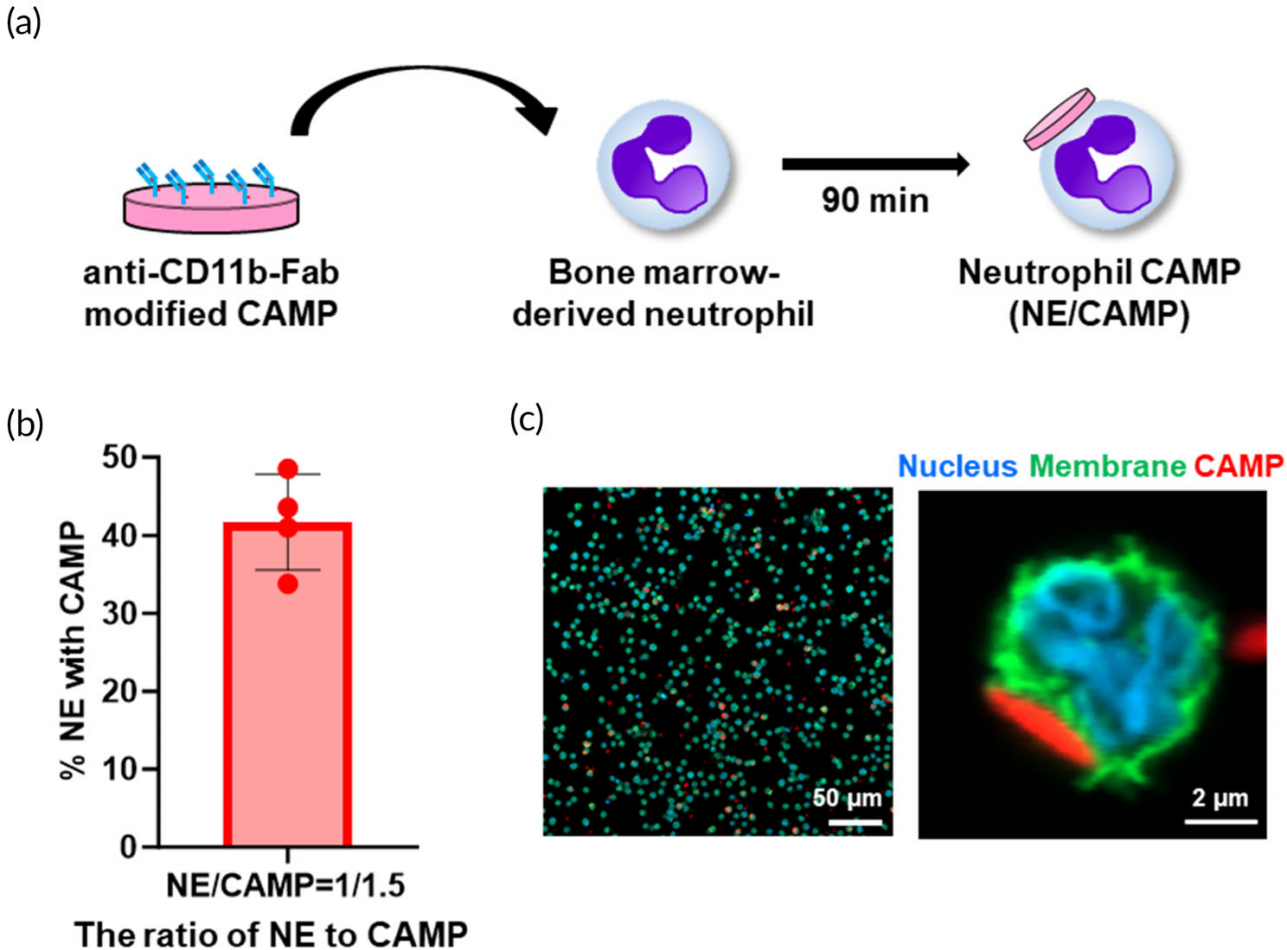
Preparation of neutrophils carrying CAMPs (NE/CAMPs). (a) Scheme for the preparation of NE/CAMPs through incubation of anti‐CD11b‐Fab conjugated CAMPs and bone marrow‐derived fresh NEs. (b) Attachment efficiency of CAMPs to NEs represented as % of NEs with at least one CAMP. (c) Confocal images of NEs after incubation with CAMPs. The right image indicates representative NE with an attached CAMP.

We prepared CAMPs composed of PLGA and PLGA‐PEG‐maleimide at a weight ratio of 2:1 and modified them with Fab fragment of anti‐CD11b antibody (aCD11b‐Fab) through a click reaction between maleimide groups displayed on CAMPs and thiol groups of aCD11b‐Fab. The design of CAMPs in this study was similar to that reported in our earlier study. That is, the average diameter of CAMPs was 6.04 μm, the average thickness was 625 nm, and the average aspect ratio was 10.04.^26^ Murine NEs were obtained from freshly isolated bone marrow cells by immunomagnetic negative selection, and incubated with aCD11b‐Fab‐modified CAMPs for 90 minutes at a ratio of NE to CAMP of 1:1.5 (Figure 1a). The attachment efficiency of CAMPs to NEs (NEs with at least one CAMP) was 41.7% (Figure 1b). Confocal images showed that aCD11b‐Fab‐modified CAMPs attached to the membrane of NEs (Figure 1c). NEs attached with CAMPs are referred to as “NE/CAMPs” in the subsequent experiments.

### 2.2 Anti‐tumor efficacy of NE/CAMPs combined with aPD‐1 in a subcutaneous GBM model

Our previous results have demonstrated that CAMP attachment to NE significantly increases the release of myeloperoxidase (MPO) and tumor necrosis factor (TNF)‐α compared with untreated NEs showing activation and a shift towards anti‐tumor N1 phenotype in vitro.^26^ Intravenous administration of NE/CAMPs also was found to be safe and showed a significant reduction in tumor burden in mice bearing mammary carcinoma or melanoma. Based on these data, we evaluated the anti‐tumor effect of NE/CAMP in a subcutaneous GL261 mouse tumor model. The mice were intravenously injected with NE/CAMPs or NE alone twice after the average tumor volume reached over 50 mm^3^ (Figure S1a). NE administration alone tended to suppress the tumor growth compared to saline treatment, although the difference was not statistically significant. Treatment with NE/CAMPs significantly suppressed tumor growth (Figure S1b). Treatment of GL261 cells with NE/CAMPs did not show a direct cytotoxic effect in vitro (Figure S2). We hypothesized that the effect of NE/CAMPs on GL261 tumors is mediated by the modulation of immune response rather than direct cytotoxicity. Median survival significantly increased by 4 and 6 days in NE‐treated and NE/CAMP‐treated groups, respectively, compared to the saline‐treated control group (Figure S1c), whereas there was no significant difference between the groups treated with NE and NE/CAMP.

**FIGURE 2:**
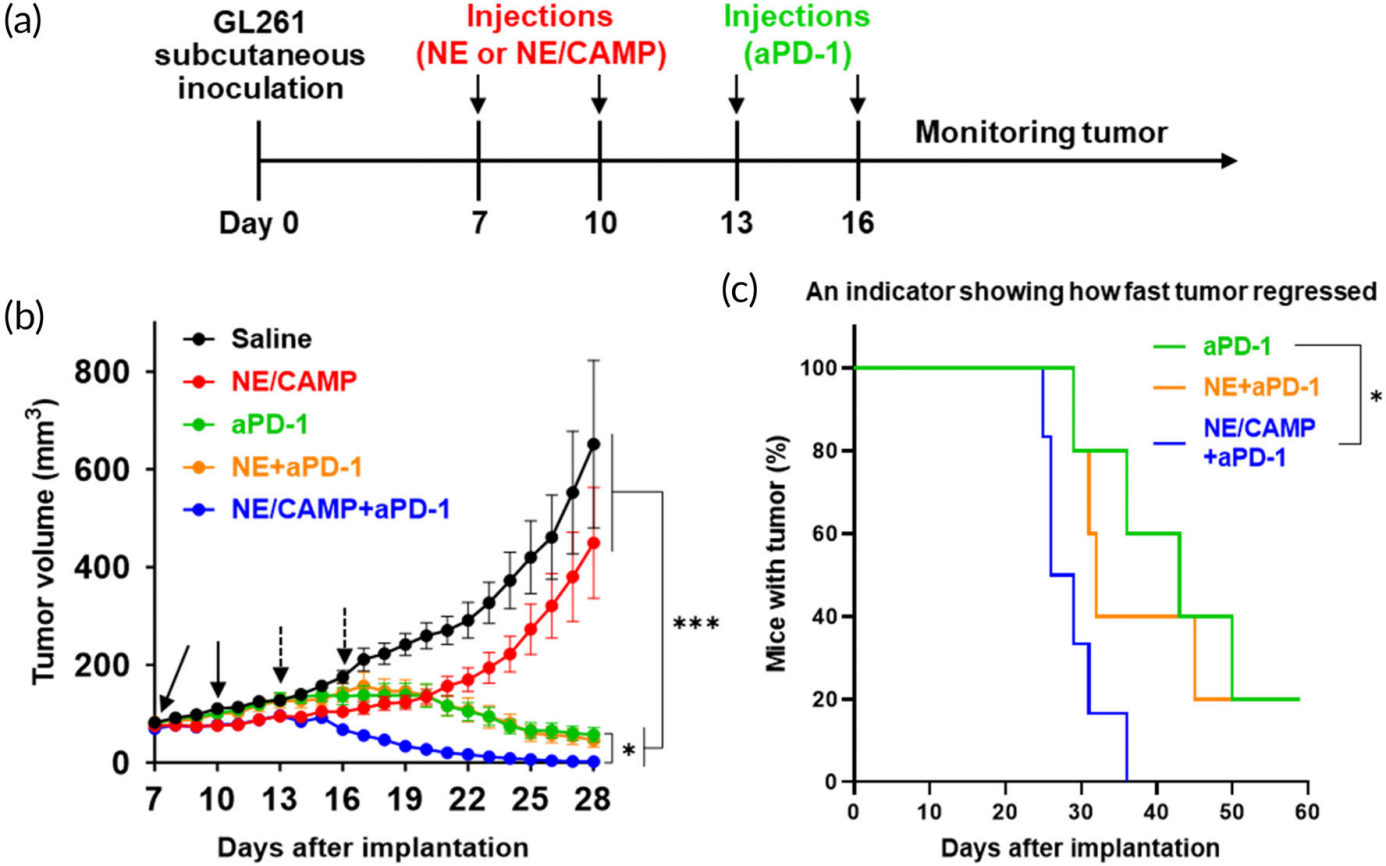
Combination effect of NE/CAMPs and aPD‐1 on subcutaneous GL261. (a) Mice bearing subcutaneous GL261 tumors (5 × 10^6^ cells/mouse) were treated with NE/CAMPs (i.v., 2 × 10^6^ NEs/injection), NEs (i.v., 2 × 10^6^ NEs/injection), or saline on 7 and 10 days after tumor inoculation. The average tumor volume reached 70–80 mm^3^ on Day 7. The mice were also treated with aPD‐1 (i.p., 100 μg/injection) on Day 13 and 16. (b) The tumor growth kinetics and (c) Fraction of mice bearing subcutaneous tumor (%) (an indicator to show how fast the tumor regressed by each treatment) monitored daily. Data are mean ± SEM (*n* = 5 for saline, NE/CAMP, aPD‐1, NE + aPD‐1, and *n* = 6 for NE/CAMP+aPD‐1). *p < 0.05, and ***p < 0.001. Black and dotted arrows in (b) indicate the treatment days of therapeutic cells and aPD‐1, respectively. Statistical differences in tumor volume (b) were analyzed by two‐way ANOVA with Sidak's multiple comparison test, and those in Mice with tumor (%) (c) were analyzed by log‐rank test.

We next assessed whether the NE/CAMP can synergistically enhance the efficacy of aPD‐1. Tumor‐bearing mice were intravenously injected with NE/CAMPs or NEs alone twice after average tumor volume reached 70–80 mm^3^, and an intraperitoneal treatment of aPD‐1 was started 3 days after the second NE/CAMP treatment (Figure 2a). Treatment with aPD‐1 alone showed good efficacy and led to significant suppression of tumor growth (Figure 2b). This may primarily be attributed to the increased immunogenicity of GL261 tumors caused by ectopic inoculation since subcutaneously implanted ectopic tumors are generally considered to be less immunosuppressive TME compared with orthotopic tumors,^33^, ^34^ which led to high therapeutic response to the aPD‐1 treatment. Even faster tumor regression was observed upon combining aPD‐1 with the NE/CAMP treatment (Figure 2b). The NE/CAMP+aPD‐1 group showed a rapid therapeutic response after the first aPD‐1 administration, and complete tumor regression was observed in 50% of mice on Day 28 after tumor inoculation (Figure 2c). No significant change in the tumor progression kinetics was observed when aPD‐1 treatment was combined with untreated NEs compared to aPD‐1 treatment alone (Figure 2b). All tumors in NE/CAMP+aPD‐1 group completely regressed by Day 40, while only 40% and 60% tumor regression were observed in the groups of aPD‐1 alone and NE+aPD‐1 on Day 40, respectively (Table 1, Figure S3).

**TABLE 1:** Percentage of tumor regression (reported as the proportion of mice with completely regressed tumors over total number of mice in that group) on each day after tumor implantation by the treatment with NE/CAMPs and aPD‐1.

	aPD‐1	NE+aPD‐1	NE/CAMP+aPD‐1
Day 28	0/5 (0%)	0/5 (0%)	3/6 (50%)
Day 40	2/5 (40%)	3/5 (60%)	6/6 (100%)
Day 60	4/5 (80%)	4/5 (80%)	6/6 (100%)

To investigate the persistence of immune responses induced by the NE/CAMP+aPD‐1 combined treatment, we carried out a tumor rechallenge study. Mice that showed complete regression by Day 60 were inoculated again with subcutaneous GL261 tumors on the opposite flank on Day 61 (Figure S4a). No additional treatment was administered. Age‐matched healthy mice were inoculated with subcutaneous GL261 tumors as controls. The tumor volume of the non‐treated healthy mice gradually increased (Figure S4b). The tumors of the rechallenged mice also grew until around 7 days after the second tumor implantation; however, all of the rechallenged tumors eventually regressed. Notably, the maximum tumor volume of the mice in the NE/CAMP +PD‐1 group was significantly smaller than other groups on Days 7 and 14 after tumor rechallenge (Figure S4c,d). Consequently, the time required for complete regression of secondary tumors was significantly lower in NE/CAMP +aPD‐1 group than in other groups (Figure S4e), indicating that the combination treatment induced a stronger long‐term anti‐tumor immune response against subcutaneously inoculated GL261.

### 2.3 Therapeutic efficacy of NE/CAMP+aPD‐1 combination in the orthotopic GBM model

**FIGURE 3:**
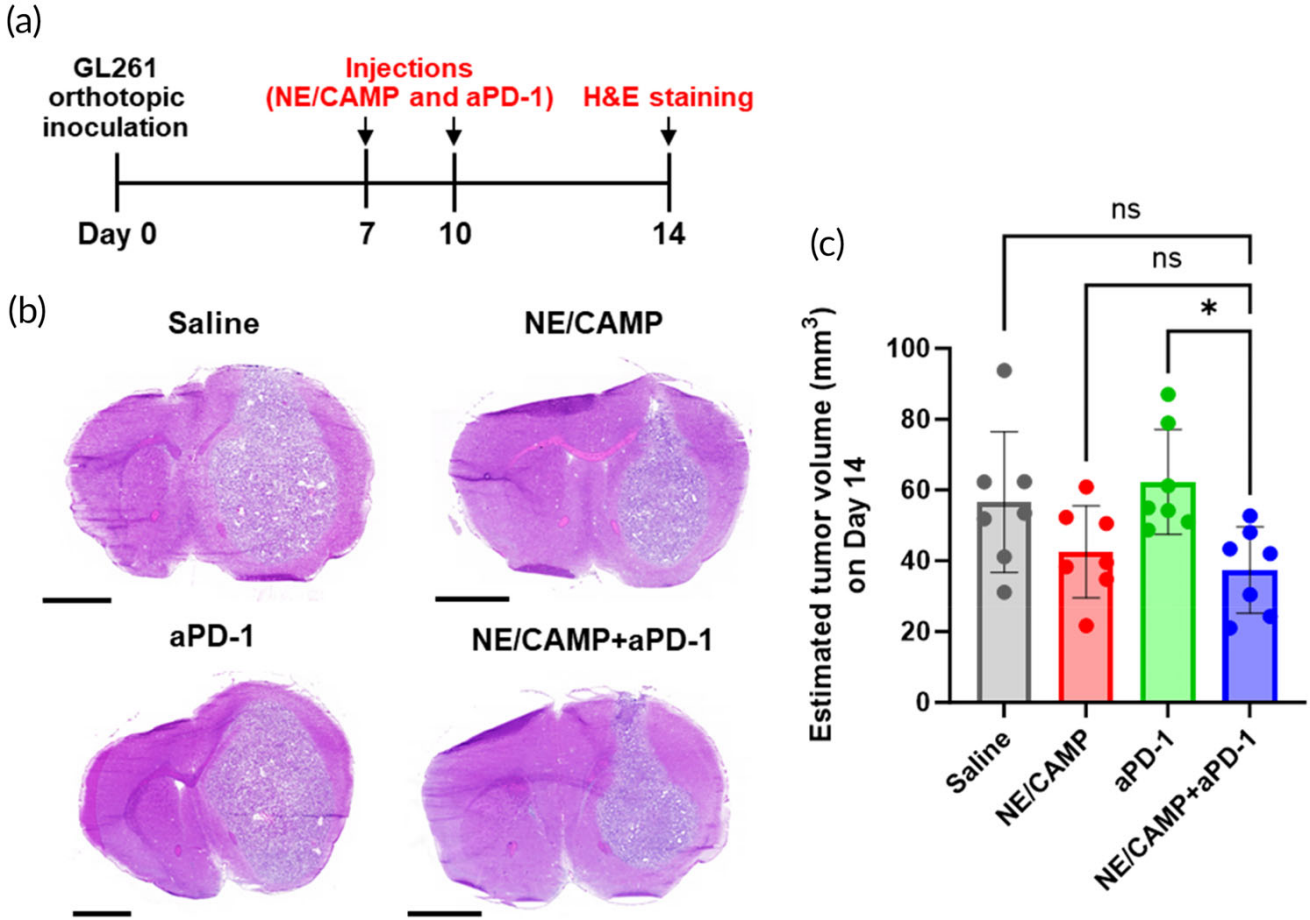
Therapeutic efficacy of the combination of NE/CAMPs and aPD‐1 on orthotopic GBM model mice. (a) Dosing regimen for the treatment of mice bearing orthotopic GL261 GBM model. The mice were treated with both NE/CAMPs (i.v., 3 × 10^6^ NEs/mouse/injection) and aPD‐1 (i.p., 100 μg/mouse/injection) twice on 7 and 10 days after tumor injection. On day 14, the brains were harvested, and 10‐μm frozen brain sections were stained with H&E. (b) Representative images of the frozen brain section stained with H&E. Scale bars: 2 mm. (c) Estimated tumor volume (mm^3^) calculated using maximum cross‐sectional area analyzed from H&E images using NIH Image J. Data are mean ± SD (*n* = 7 for each group). Statistical differences were analyzed by one‐way ANOVA with Tukey's multiple comparison tests. *p < 0.05.

Since the combination of NE/CAMPs and aPD‐1 exhibited a potent anti‐tumor effect on GBM cells in vivo in the subcutaneous GL261 model, we investigated the potency of NE/CAMP+aPD‐1 combination therapy in an orthotopic GL261 GBM mouse model. The orthotopic model recapitulates several key features of glioblastoma, such as the presence of blood–brain barrier and cold tumor microenvironment, that rendered traditional immunotherapies less efficacious. Mice were treated with both NE/CAMPs (i.v.) and aPD‐1 (i.p.) twice on days 7 and 10 after tumor implantation. Brains were harvested on Day 14 to evaluate intracranial tumor size by hematoxylin and eosin (H&E) staining (Figure 3a). Brain tumors were observed in the right brain hemisphere (Figure 3b). Tumor volumes were calculated based on the maximum tumor area analyzed with an image software NIH ImageJ.^35^, ^36^ Treatment with NE/CAMP alone tended to decrease the tumor size compared to the saline‐treated group whereas the aPD‐1‐treated group exhibited no effect (Figure 3b,c). The combined treatment of NE/CAMPs and aPD‐1, however, significantly suppressed tumor growth in comparison to aPD‐1 alone group (40% decrease in tumor volume), and it exhibited the highest tumor suppression effect.

Therapeutic efficacy was also evaluated by monitoring the survival rate. Mice were treated three times with NE/CAMPs and aPD‐1 every 3 days starting from Day 7 (Figure S5a). aPD‐1 treatment alone did not exhibit any improvement in survival compared with the saline group except for one mouse (Figure S5b). Median survival in control and aPD‐1‐treated mice was 18 days. The median survival for mice treated with NE/CAMP alone and NE/CAMP+aPD‐1 was extended to 19.5 and 20 days, respectively. Mice treated with NE/CAMP+aPD‐1 exhibited a significant improvement in survival compared with the saline group, as determined by log‐rank test (*p* = 0.0073). Although complete tumor regression was not observed in the orthotopic model, primarily due to strong physical and immunological barriers, the treatment with NE/CAMPs and aPD‐1 exhibited improved therapeutic efficacy against GBM.

### 2.4 Cerebral distribution of NE/CAMPs into the GBM region

**FIGURE 4:**
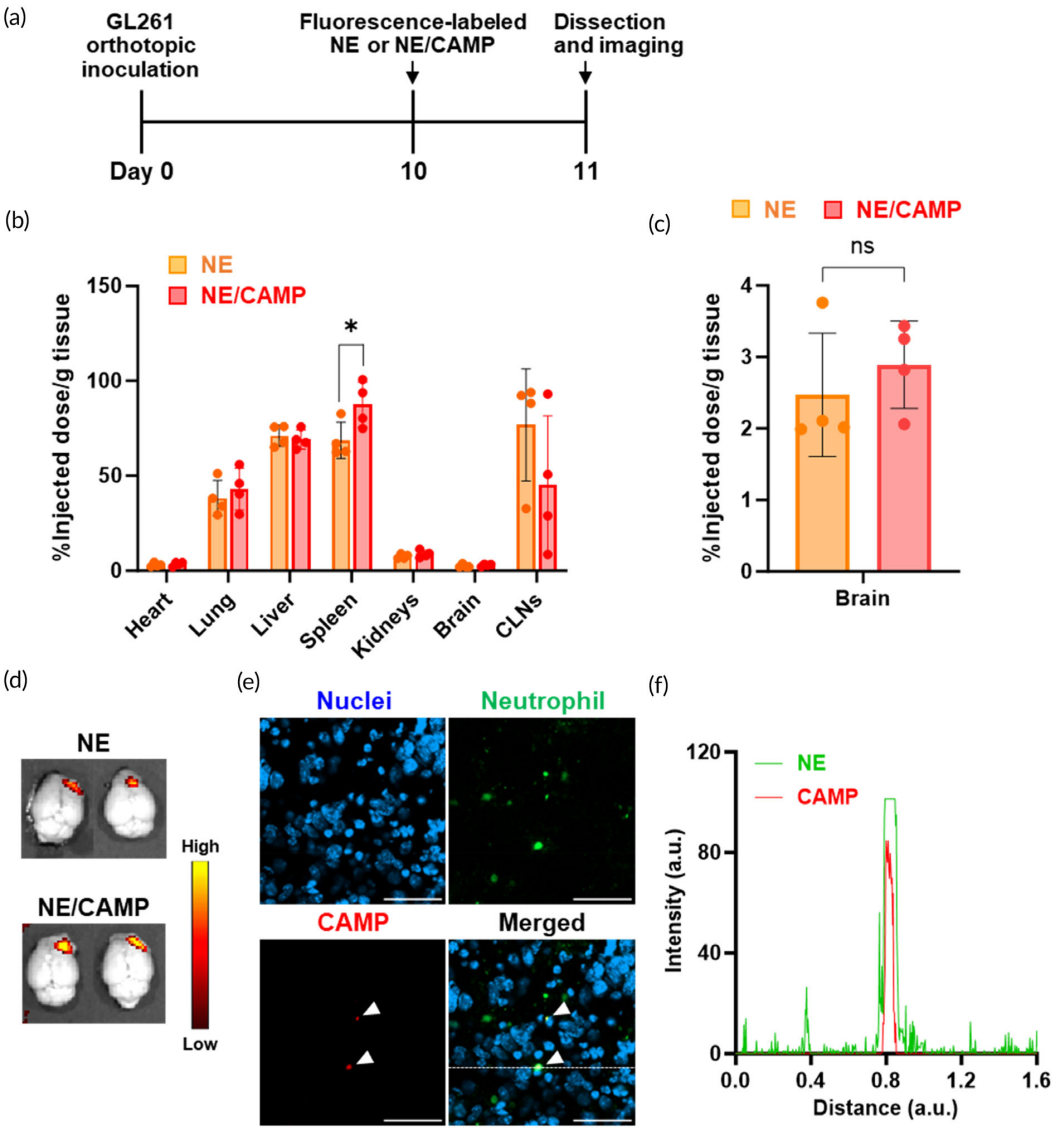
Biodistribution of NE/CAMPs in orthotopic GL261 mice. (a) GL261 cells (1 × 10^6^ cells/mouse) were orthotopically injected into the brain, and VivoTrack680‐labeled NEs or NE/CAMPs (2 × 10^6^ NEs/mouse) were intravenously injected into the mice 10 days after tumor implantation. (b) Accumulation of adoptively transferred neutrophils represented as the fluorescence intensity of each organ normalized to both the total intensity acquired from all the organs and the weight of the organs (%injected dose / g tissue) analyzed using IVIS at 24 h after intravenous administration of the samples. Data are mean ± SD (n=4). *p<0.05 (c) Accumulation of adoptively transferred neutrophils in the brain Data are mean ± SD (*n* = 4). (d) The representative images of fluorescence in the brain of the groups of NE (upper) and NE/CAMP (lower). (e) The fluorescence images of the brain tumor obtained by confocal laser scanning microscopy. Blue, green, and red indicate nuclei (DAPI), Ly6G‐positive NEs (Alexa488), and CAMPs (Rhodamine B), respectively. The white arrows in the images indicate CAMPs derived from Rhodamine B fluorescence. Scale bars: 100 μm. (f) The graph of intensities (NE and CAMP) versus distance at one dotted line in the merged image in (e) representing the co‐localization of CAMPs and NEs.

To assess the mechanism of the efficacy of NE/CAMP + aPD‐1, we performed a biodistribution study of NE/CAMPs to investigate whether NE/CAMPs reached the GBM region and to examine the influence of CAMP attachment to NEs on their in vivo trafficking. Upon intravenous administration, NEs and NE/CAMPs mainly accumulated in the liver, spleen, and lungs 24 h after intravenous injection (Figure 4a,b and S6). Regardless of CAMP treatment, the accumulation of adoptively transferred neutrophils in major organs was similar, except spleen, where the accumulation of NE/CAMPs was significantly higher than NEs (Figure 4b). Although the detailed cause is still unclear, CAMPs attached to the NEs might slightly affect the biodistribution in the spleen. Treatment with CAMPs did not affect the ability of NEs to accumulate in the brain or in the cervical lymph nodes (CLNs, draining lymph nodes for GBM; Figure 4c). Importantly, accumulation of both NEs and NE/CAMPs in the brain was observed to be localized in the right hemisphere, around the GBM region (Figure 4d). Moreover, imaging using confocal laser scanning microscopy revealed that CAMPs and NEs were colocalized in the GBM tissue (Figure 4e,f), indicating that CAMPs could remain attached to the adoptively transferred NEs as they infiltrated into the brain. These results indicate that CAMP attachment did not have a substantial impact on the in vivo fate of NEs and that NE/CAMPs accumulated into the GBM tissue by passing through the BBB owing to the intrinsic migration ability of NEs.

### 2.5 Induction of systemic anti‐tumor immune response by NE/CAMP+aPD‐1

**FIGURE 5:**
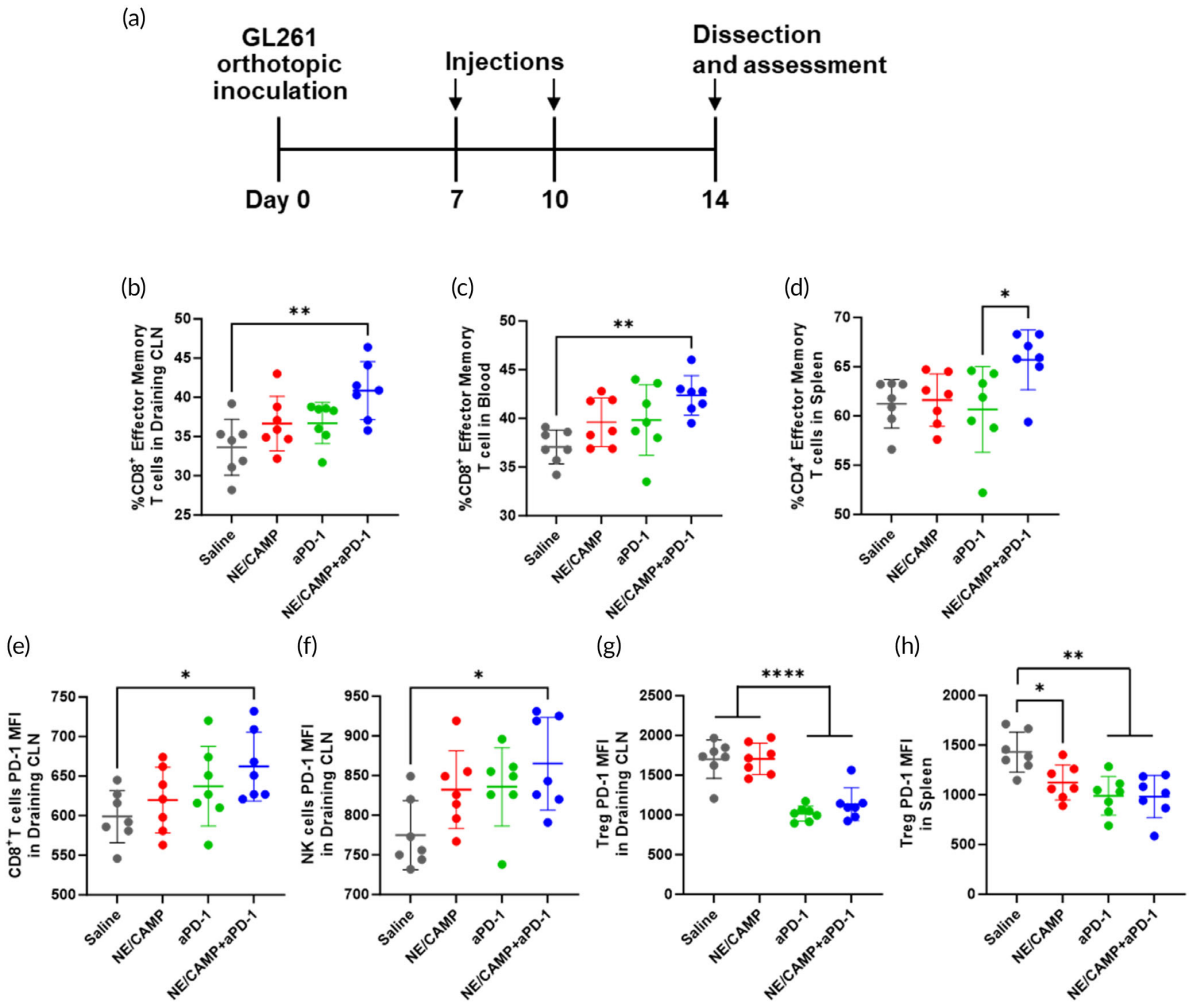
Activation of systemic immune responses by combination treatment in orthotopic GL261 mice. (a) GL261 cells (1.0 × 10^5^ cells) were intracranially injected into C57BL/6 mice to inoculate orthotopic GBM tumors into mice. The mice were treated with NE/CAMPs (i.v., 3 × 10^6^ NEs/mouse/injection), aPD‐1 (i.p., 100 μg/mouse/injection), or saline at 7 and 10 days after tumor implantation. On day 14, blood, spleen, and draining CLNs were harvested and processed into single‐cell suspensions to analyze immune profiles by flow cytometry. (b) The proportion of CD8^+^ effector memory T cells (CD45^+^ CD3^+^ CD8^+^ CD44^+^ CD62L^−^). (c) The proportion of CD4^+^ effector memory T cells (CD45^+^ CD3^+^ CD4^+^ CD44^+^ CD62L^−^). (d and e) PD‐1 expression levels on CD8+ T cells (d) and NK cells (e) in draining CLNs. (f‐h) PD‐1 expression levels on NK cells (f) in draining CLNs, and Treg (CD45^+^ CD3^+^ CD4^+^ FOXP3^+^) in draining CLNs (g) and spleen (h). Data are mean ± SD (*n* = 7 for each group). Statistical differences were analyzed by one‐way ANOVA with Tukey's multiple comparison tests. *p < 0.05, **p < 0.01, and ****p < 0.0001.

Due to the inability of NE/CAMPs to induce direct cytotoxicity against GBM cells, we hypothesized that the superior efficacy of NE/CAMP treatment may be due to the modulation of local and systemic anti‐tumor immune response. To assess this, we performed immunophenotyping of immunologically important organs such as the spleen, CLNs, brain, and blood. These organs were harvested from the mice bearing orthotopic GBM tumors 4 days after the second treatment with NE/CAMPs and aPD‐1, that is, Day 14 after tumor implantation (Figure 5a). Single‐cell suspensions obtained from these organs were analyzed via flow cytometry to evaluate the proportions and phenotypes of various immune cell populations (Figures S9 and S10). A significant increase in the population of CD8^+^ effector memory T cells was observed by the NE/CAMP+aPD‐1 treatment in draining CLNs (21% increase) and blood (14% increase) (Figure 5b,c). In the spleen, the NE/CAMP +aPD‐1 treatment resulted in a 7% increase in the proportion of CD4^+^ effector memory T cell population (Figure 5d). Expression of PD‐1, one of the markers expressed on activated T cells upon immune responses, in CD8^+^ T cells significantly increased (11% increase) in draining CLNs in NE/CAMP+aPD‐1 group compared with the control group (Figure 5e). PD‐1 expression of NK cells in draining CLNs was also significantly increased (12% increase) upon combination treatment (Figure 5f). PD‐1 expression in regulatory T cells (Treg) significantly decreased in the NE/CAMP+aPD‐1 (33% reduction) and aPD‐1 alone (40% reduction) groups in the draining CLNs (Figure 5g). PD‐1 expression in regulatory T cells also decreased in the spleen across NE/CAMP+aPD‐1, NE/CAMP, and aPD‐1 alone groups (Figure 5h). The NE frequency remained unchanged in blood, spleen, and draining CLNs in the NE/CAMP and combination treatments (Figure S7), indicating that adoptively transferred NEs were eliminated within 4 days after the treatment and did not affect the overall NE population in the body.

To evaluate immunophenotype in the brain tumor tissues, immunofluorescence analysis was performed using frozen brain tissues prepared from brain samples obtained on Day 14 after tumor implantation. The population of F4/80‐positive tumor‐associated macrophages was unchanged by each treatment (Figure S8a). The expression of representative markers of pro‐inflammatory (M1) and anti‐inflammatory (M2) macrophages, namely inducible nitric oxide synthase (iNOS) and CD206, was also unchanged compared to saline and each treatment group, respectively (igure S8b,c). The percentage of FOXP3‐positive Treg significantly decreased (74% reduction) by NE/CAMP+aPD‐1 treatment compared with the saline group (Figure S8d,f), suggesting that the combination therapy could reduce the accumulation of suppressive T cells in the GBM. On the other hand, the proportion of CD8+ T cells decreased significantly in the NE/CAMP+aPD‐1 treatment compared with the saline group (Figure S8e), suggesting a limited infiltration of effector cells at the time of analysis. Based on these results, we posit that induction of systemic anti‐tumor immune response and suppression of Treg activities could contribute to the anti‐tumor effect of the NE/CAMPs+aPD‐1 combination therapy against GBM while the limited efficacy of the combination therapy may be a result of limited modulation of local immunological milieu.

## 3 DISCUSSION

NEs are the most abundant blood‐circulating leukocytes in humans and they are involved in the pathology of several cancers.^37^ A large body of evidence has demonstrated the ability of NEs to directly kill cancer cells by releasing certain mediators, such as reactive oxygen species and MPO, and antibody‐dependent cellular cytotoxicity (ADCC).^38^ Also, activated NEs can stimulate anti‐tumor immunity via recruiting activated immune cells by chemokines and inflammation cytokines.^39^ NEs, despite having such promising anti‐tumor aspects, have not been employed as a cell of choice for adoptive cell therapy. We have recently proposed the use of NEs for cancer immunotherapy via a drug‐free material‐based approach.^26^ Adoptive transfer of activated NEs via surface attachment of disk‐shaped micro‐patches “CAMPs” induced a robust anti‐tumor systemic immune response, resulting in suppression of tumor growth and synergistic anti‐tumor effects in case of combination with ICIs in melanoma.^26^ The efficacy of ICIs in treating GBM is limited due to the highly immunosuppressive environment within the brain as well as throughout the body.^13^, ^20^ Therefore, new treatments to improve immunosuppressive conditions of GBM and to augment the efficacy of ICIs are highly desired. For this purpose, the present study investigated the adoptive transfer of NE/CAMPs to generate a robust immune response against GBM and then increase the efficacy of ICI treatment.

Studies in the subcutaneous GL261 model revealed that treatment with NE/CAMPs exhibited a higher anti‐tumor effect than NE alone, and more rapidly regressed the tumor when combined with aPD‐1 (Figures 2 and S1). We previously demonstrated that NE/CAMPs, but not NEs alone, induce systemic immune activation, and this activation is important for the anti‐tumor effect by NE/CAMPs.^26^ No significant difference observed in survival in mice bearing subcutaneous GBM in response to the treatment with NE alone and NE/CAMP groups (Figure S1) can possibly be attributed to either insufficient activation of NEs by CAMPs or suboptimal dosing regimen. In addition, the ability of NE/CAMPs to induce direct cytotoxicity may be different for different cancer cells. NE/CAMPs were ineffective in killing GL261 cells directly in this study, but not against 4T1‐Luc in our previous study. This prompts further optimization of CAMP design and dose optimization specifically for GBM to obtain superior therapeutic responses as parts of future investigation. NE‐mediated direct cancer cell‐killing effect has been demonstrated to be enhanced in IFN‐γ‐dependent manner in vitro.^40^ Another in vitro study revealed that NE treatment with a cocktail consisting of TNF‐α, CD40 agonist, and a tumor‐specific antibody showed increased tumor‐killing activity in human tumor cells through ADCC.^41^ Based on these findings, combination with other cytokines such as IFN‐γ as well as tumor cell‐specific antibodies may enhance the direct cytotoxic effect of NE/CAMP against GBM cells in vitro.

Previously we also demonstrated synergistic effects of NE/CAMP and ICIs in treating melanoma in mouse models.^26^ The results presented here extend previous findings to a glioblastoma tumor model (Figure 2). In the orthotopic GL261 model, the NE/CAMP+aPD‐1 combination systemically activated T cell responses (Figure 5). Activation of T cell immune response is crucial for subsequent memory T cell responses.^42^ Based on these findings, we posit that the NE/CAMP+aPD‐1 combination induces potent and long‐term systemic immune activation, resulting in a superior anti‐tumor effect compared with other groups and induces complete regression of subcutaneously implanted GL261.

The results of immunophenotyping analyses in the GL261 orthotopic model indicated that the NE/CAMP+aPD‐1 combination treatment activated T cell‐ and NK cell‐mediated systemic immune responses, and decreased Treg activation in both spleen and CLNs as well as reduced the Treg population in the tumor tissue (Figure 5 and S8). Moreover, the NE/CAMP+aPD‐1 combination treatment significantly suppressed brain tumor growth compared to the treatment with aPD‐1 alone (Figure 3), which led to a significant improvement in survival (Figure S5). For the optimal cancer immunity cycle in GBM, it was recently reported that CLNs are involved in immune cell trafficking from the central nervous system into CLNs via meningeal lymphatic vessels and that T cell priming and activation occur in draining CLNs, which are systemically suppressed under GBM conditions.^43^ Further, the therapeutic efficacy of aPD‐1 was reported to increase by activation of those lymphatic systems,^44^, ^45^ indicating the importance of draining CLNs in GBM therapy. Indeed, in clinical trials of dendritic cell (DC) vaccines for GBM, a few trials employ intradermal injection close to CLNs (NCT04888611, NCT04388033) and intranodal injection to CLNs (NCT00323115) to improve DC migration into draining CLNs and to enhance subsequent immune responses, although detailed data are awaited. Considering these findings, we posit that activation of a systemic anti‐tumor immune response including draining CLNs and suppression of Treg activities could lead to an effective anti‐tumor effect of the NE/CAMPs and aPD‐1 combination against GBM.

Although the combination therapy with NE/CAMPs and aPD‐1 showed significant therapeutic improvement in orthotopic GBM, complete tumor regression could not be achieved. Also, the combination of aPD‐1 with NE/CAMP only marginally increased therapeutic response compared with NE/CAMP alone. Insufficient improvement in the immunosuppressive TME, especially the phenotype of tumor‐associated macrophages (Figures S8a–c) and reduction in the amount of CD8+ T cells (Figures S8e) at the time of analysis, are considered to be the reasons why the anti‐tumor effect of NE/CAMP+aPD‐1 was only slightly increased by aPD‐1 combination compared with NE/CAMP alone. Additionally, GBM induces a distinctive systemic immunosuppressive environment and a locally cold brain TME, further hindered by the BBB and blood‐tumor barrier.^14^ Exhaustion of tumor‐infiltrating immune cells such as CD8^+^ T cells and NK cells is another major hurdle for achieving successful immunotherapy against GBM.^46^ Hence, it is necessary to modulate both systemic and local TME and prevent immune cell exhaustion to achieve better therapeutic responses against GBM. Biological cues such as lipopolysaccharides, interferon‐beta (IFN‐β), and transforming growth factor‐β (TGF‐β) inhibitors have been reported to induce NE phenotype switch from a pro‐tumor N2 to anti‐tumor N1 type.^47^ Indeed, intra‐tumoral injection of NE activators brought about tumor suppression in an NE‐dependent manner,^41^ although continuous stimulation is needed to keep activated N1 type in vivo. Our previous studies have reported successful encapsulation of cytokines (IFN‐γ and interleukin‐4) and small molecules (dexamethasone) into CAMPs, and achieved persistent immunomodulation of immune cells, namely macrophages and monocytes, by surface attachment of CAMPs encapsulating those immunomodulators.^48^, ^49^ Intratumoral administration of IFN‐γ loaded CAMP‐adhered macrophages achieved polarization of both adoptively transferred and tumor‐associated macrophages into anti‐tumor type, resulting in reducing metastasis and tumor burden of 4 T1 breast cancer.^49^ Also, adoptive transfer of monocytes carrying CAMPs encapsulating IL‐4 and dexamethasone enabled persistent conversion of their phenotypes to anti‐inflammatory type, which allowed for modulation of both the brain and systemic immune responses and treatment of multiple sclerosis.^48^ In the present study, we found that CAMP attachment does not affect the migration ability of NEs into the GBM region and the delivered CAMP retained on NEs in the brain (Figure 4). With further research focused on optimization and encapsulation of NE activators, CAMP‐adhered NEs may provide a potent tool in treatment of GBM.

NEs, due to their intrinsic biology, offer unique advantages and challenges to the translation. The MHC‐independent activity of NEs offers a choice of a wider donor population in the form of allogeneic transfer. However, due to the short lifespan of NEs, it is not advised to cryopreserve NEs for long durations without affecting the recovery. For this reason, granulocyte transfusion therapies are traditionally performed within 6 h of allogenic cell harvest.^50^ The NE/CAMP approach described here activates and polarizes NEs within less than 2 h without employing genetic engineering or biochemical stimulation of the harvested NEs. Hence, it is possible to utilize an extra‐corporeal device to modify NEs and infuse them back into the patients within the recommended 6 h timeframe. The CAMP‐based engineering approach may advance the development of NE‐based cancer immunotherapy including GBM.

## 4 CONCLUSION

Our results demonstrate the therapeutic potential of adoptive transfer of CAMP‐activated NEs in combination with aPD‐1 for the treatment of GBM. The NE/CAMP+aPD‐1 combination treatment showed superior anti‐tumor effect compared with each treatment alone and completely regressed subcutaneously implanted GBM as well as rechallenged one. The intracerebral accumulation of NE/CAMP was observed in tumor of orthotopic GBM model mice, indicating that NE's migration ability into the brain was not affected by CAMP attachment. Also, the combination treatment activated a systemic anti‐tumor immune response in the orthotopic GBM mouse model and improved therapeutic efficacy of aPD‐1. Taken together, the present study suggests that adoptive transfer of NE/CAMPs could be useful for the treatment of GBM by increasing the efficacy of combined ICIs.

## 5 METHODS

### 5.1 CAMP preparation

CAMPs were prepared by spin coating and subsequent microcontact printing as reported previously.^51^ Briefly, silicon wafers were prepared with patterned photoresist in an array of 6 μm holes. Polydimethylsiloxane (PDMS; Sylgard 184 Silicone Elastomer Kit, Dow) templates with 6‐μm pillars in diameter were fabricated by casting from the patterned silicon wafers. An 8% w/v% polymer solution composed of PLGA (7 to 17 kDa; Resomer 502H, Sigma‐Aldrich) and PLGA‐PEG‐Maleimide (10 kDa PLGA and 5 kDa PEG; Nanosoft Polymers) at a weight ratio of 2:1 was prepared in dichloromethane. For fluorescence labeling of backpacks, PLGA‐rhodamine B (10 kDa PLDA; Nanosoft Polymers) was mixed into the initial polymer solution at a ratio of 100:1 (non‐fluorescent to fluorescent PLGA). The PLGA solution was spin‐coated onto PDMS templates at 1500 rpm for 35 s, drying at room temperature for over 1 h. Polyvinyl alcohol (PVA; 13 to 23 kDa, 87%–89% hydrolyzed, Sigma‐Aldrich) coated dishes were prepared by adding 3 w/v% PVA solution onto 100‐mm Petri dishes and then drying overnight at 65°C. The spin‐coated PDMS stamps were pressed onto preheated PVA‐coated dishes to transfer the CAMPs onto the dishes. The printed CAMPs on the dishes were stored at −20°C before use. The CAMPs were harvested by washing the PVA‐coated dishes twice with PBS and then filtered through a 40‐μm cell strainer to eliminate debris, followed by centrifugation at 2000 *g* for 8 min to collect and enrich CAMPs for subsequent experiments.

### 5.2 Antibody modification of CAMPs

aCD11b‐Fab was prepared as previously reported.^26^ Briefly, rat anti‐mouse CD11b IgG (BioLegend) was digested with a Pierce F(ab')_2_ Preparation Kit (Thermo Fisher Scientific), and the digested protein was purified using affinity column with AminoLink Plus Immobilization Kit (Thermo Fisher Scientific) in accordance with manufacturer's recommended protocol by using Goat anti‐rat IgG Fc (Thermo Fisher Scientific) as the coupling protein. Purified F(ab')2 fragments were obtained by elution through the affinity column, and the fragments were enriched to at least 1 mg/mL by ultrafiltration (Amicon Ultra‐4 MWCO 10K; Millipore Sigma). The enriched fragments were then reduced with dithiothreitol (DTT; 1.8 mM in 10 mM EDTA) for 20 minutes at room temperature, followed by desalting with Zeba Spin Desalting Columns (7 K MWCO; Thermo Fisher Scientific) three times for DTT removal. Surface modification of CAMPs with the aCD11b‐Fab was performed in PBS for 45 min at room temperature before attachment with NEs by incubating CAMPs with a significant excess amount of aCD11b‐Fab.

### 5.3 Attachment of CAMPs to neutrophils

Female 6‐7‐week‐old C57BL/6 mice were purchased from Charles River Laboratories. Experimental protocols using animals were reviewed and approved by the Institutional Animal Care and Use Committee (IACUC) at Harvard University. The mice were maintained under 12 h light/12 h dark cycles with free access to water and food.

NEs were isolated from murine bone marrow by immunomagnetic negative selection. After C57BL/6 mice were euthanized by overdose carbon dioxide inhalation, two bones (tibia and femur) and one bone (humerus) from each hind and front limb were dissected, respectively. Epiphyses of each bone were cut, bones were then flushed with EasySep Buffer (STEMCELL Technologies), and bone marrow‐derived cells were filtered through 100‐μm cell strainer into 50‐mL tube, followed by centrifugation at 300 g for 10 min. The collected cells were resuspended in EasySep Buffer, and NEs were isolated with EasySep Mouse Neutrophil Enrichment Kit (STEMCELL Technologies) according to the manufacturer's manual. NEs and CAMPs were resuspended in RPMI1640 medium supplemented with 1% penicillin/streptomycin (Pen Strep) and 10% fetal bovine serum (FBS) at 20 × 10^6^ cells/mL and 30 × 10^6^ particles/mL, respectively. CAMP attachment was performed by mixing equal volume (50 μL) of each suspension in a low‐binding 96‐well U‐bottom plate and subsequent incubation for 90 min at 37°C in a 5% CO_2_ incubator. CAMP attachment was quantified by flow cytometry and imaged by confocal laser scanning microscopy. To perform confocal imaging, NE/CAMPs were added onto poly‐L‐lysine (PLL)‐coated glass slides and incubated for 20 min at room temperature. The cells were fixed in Fixation Buffer (BioLegend) for 10 min and permeabilized with Perm/Wash Buffer (BD Biosciences) for 3 min, followed by staining with ActinGreen 488 Readyprobes (Thermo Fisher Scientific) and DAPI for 15 min. After mounting the cells with Prolong Glass (Thermo Fisher Scientific), fluorescence of CAMPs, cells, and nuclei was observed with a confocal laser scanning microscope LSM 900 (Zeiss).

### 5.4 Therapeutic study in subcutaneous GL261 tumor model

All animal studies were performed according to the protocols approved by the Institutional Animal Care and Use Committee of Harvard University.

GL261 mouse glioblastoma cell line was obtained from the National Cancer Institute. GL261 cells were cultured in Dulbecco's Modified Eagle's Medium (DMEM; Cytiva) supplemented with 1% Pen Strep and 10% FBS at 37°C in a 5% CO_2_ incubator.

GL261 cells (3 or 5 × 10^6^ cells/mouse) were subcutaneously implanted into the left posterior flank to establish a subcutaneous tumor. When average tumor volume reached 50 mm^3^, NE/CAMPs (2 × 10^6^ NEs/mouse/injection), NEs (2 × 10^6^ NEs/mouse/injection), or saline were intravenously administered into the mice twice 3 days apart. For combination experiments with aPD‐1, the treatment with NE/CAMPs, NEs, or saline was performed when the average tumor volume reached 70–80 mm^3^ (Day 7) twice 3 days apart. On Day 13 and 16, 100 μg of aPD‐1 (BioXcell; Clone: 29F.1A12) was intraperitoneally injected into the mice of the combination groups. Tumor size was measured every day with a caliper and tumor volume was calculated according to the following formula: 0.5 × *a* × *b* ^2^ (*a*, largest diameter; *b*, smallest diameter). The mice were euthanized if the largest tumor diameter exceeded 15 mm. The dose of NEs and the number of injections were determined based on our previous report, in which NE/CAMP treatment (2 × 10^6^ NEs/injection) twice 3 days apart significantly decreased tumor burden compared with saline and neutrophil alone groups in 4 T1 orthotopic breast cancer‐bearing mice and B16F10 melanoma‐bearing mice.^26^

### 5.5 WST‐8 assay

GL261 cells (2 × 10^4^ cells/well) were seeded onto 96‐well plate and incubated overnight at 37°C in a 5% CO_2_ incubator. The medium was changed from DMEM (1%P/S and 10%FBS) to RPMI (1%P/S and 10%FBS), and the GL261 cells were treated with NE/CAMPs, NE, or CAMPs at a ratio of 5:1, 2.5:1, and 1:1 (Number of NEs or CAMPs to that of GL261). After 24‐h incubation, the media containing each sample was removed, and the cells were washed with PBS (−) and cultivated in free DMEM (1% P/S without FBS) for additional 24 h. At 48 h after the addition of each sample, the GL261 cells were washed with PBS and incubated with WST‐8 working solution composed of 10% Cell Counting Kit‐8 reagent (Boster Bio) in DMEM (1%P/S without FBS) for 1 h at 37°C in accordance with the manufacturer's instructions. The viable cells were determined by measuring absorbance at 450 nm using a microplate reader.

### 5.6 Therapeutic study in orthotopic GL261 tumor model

GL261 cells (1 × 10^5^ cells in 4 μL saline) were intracranially inoculated at 2 mm right, 1 mm anterior to the right side of the bregma and 3 mm deep into the brain of female 6‐7‐week‐old C57BL/6 mice. 7 and 10 days after the inoculation, mice were treated with saline or NE/CAMPs (3 × 10^6^ NEs/injection) intravenously. The dose of NEs was increased from 2 × 10^6^ in subcutaneous studies to 3 × 10^6^ NEs/injection with the aim to increase the therapeutic response in the orthotopic model. Some mice also received a 100 μg dose of aPD‐1 intraperitoneally. On day 14 mice were humanely euthanized and brains were harvested. Harvested brain samples were frozen in OCT compound and 10‐μm frozen sections were prepared with a cryostat. The serial 10‐μm sections were stained with hematoxylin and eosin (H&E) using a H&E staining kit (Abcam) and imaged with Zeiss Axioscan. The tumor area in the brain was assessed with an image analysis software ImageJ (NIH), and the maximum cross‐sectional area of tumor tissues was analyzed. Estimated tumor volume was determined with the following formula: tumor volume = (square root of maximal tumor cross‐sectional area)^3^ in accordance with the previous reports.^26^, ^27^

### 5.7 Biodistribution of NE/CAMPs in intracranial orthotopic GL261 tumor model

C57BL/6 mice were intracranially inoculated with GL261 cells as described in section 5.5. 10 days after intracranial inoculation, NEs or NE/CAMPs fluorescently labeled with VivoTrack 680 (Perkin Elmer) were intravenously injected into the mice. At 24 h after intravenous injection, the mice were euthanized, and major organs (heart, lungs, liver, spleen, kidneys), right superficial and deep cervical lymph nodes, and brains with tumor were harvested, followed by fluorescence imaging by in vivo imaging system (IVIS Spectrum, PerkinElmer).

After IVIS imaging, the brains with tumors were embedded in OCT compound (Sakura Finetek) and the embedded tissues were frozen using dry ice/ethanol. Then, 10‐μm frozen tumor sections were prepared with cryostat, and immunostaining for Ly6G was performed to observe NEs in the brain. Briefly, the frozen brain sections were fixed with Fixation Buffer for 10 min and blocked with 3% bovine serum albumin (Sigma‐Aldrich) in PBS for 20 min at room temperature. The sections were then incubated with Alexa Fluor 488 anti‐mouse Ly‐6G antibody (BioLegend, Clone: 1A8) overnight at 4°C. Thereafter, the sections were mounted with VECTASHIELD® Antifade Mounting Medium containing DAPI (Vector Laboratories), and imaged by confocal laser scanning microscopy to visualize colocalization of NEs and CAMPs in the GBM region.

### 5.8 Immunophenotyping study

At 7 and 10 days after intracranial GL261 inoculation, the mice were injected with NE/CAMPs (3 × 10^6^ NEs/mouse/injection, i.v.) and aPD‐1 (100 μg/mouse/injection, i.p.). At 14 days after tumor inoculation, blood, spleens, draining CLNs, and brains were harvested from euthanized mice. Single‐cell suspensions were obtained for spleens and lymph nodes by passing through 40‐μm filters in cold PBS, and those suspensions were stained with Live Dead fixable blue stain (Thermo Fisher) for 30 min at 4°C. After washing twice with Hanks' Balanced Salt solution (HBSS), the cell suspension was fixed using Fixation Buffer (BioLegend) and then incubated with anti‐mouse CD16/32 blocking antibody for 10 min on ice. The cells were incubated with an antibody cocktail (Table. 2) for 30 min at 4°C. After washing three times, the immunophenotype of the cells was analyzed using a Cytek Aurora flow cytometer (Cytek Bioscience). The obtained data were analyzed with FlowJo V10.

**TABLE 2:** Information on antibodies and fluorophores for analyses of lymphoid cells.

Marker	Fluorophore	Clone	Supplier
Viability	Live Dead Blue	‐	Thermo Fisher Scientific
CD3	AF532	17A2	Thermo Fisher Scientific
CD4	BUV496	GK1.5	BD Biosciences
CD8	BV785	53–6.7	BioLegend
CD44	PE	IM7	BioLegend
CD62L	AF700	MEL‐14	BioLegend
NK1.1	PE‐Dazzle 594	PK136	BioLegend
PD‐1	BV510	RMP1‐14	BioLegend
FOXP3	PE‐Cy5.5	FJK‐16 s	BioLegend

### 5.9 Statistical analysis

Statistical analyses were performed using GraphPad Prism 9 software. As described in each figure legend, one‐way analysis of variance (ANOVA) with Tukey post hoc test and two‐way ANOVA with Sidak's multiple comparison test were used to analyze significant differences.

## AUTHOR CONTRIBUTIONS

**Tatsuya Fukuta:** Conceptualization; methodology; data curation; investigation; validation; formal analysis; writing – original draft; writing – review and editing; visualization. **Ninad Kumbhojkar:** Conceptualization; methodology; data curation; investigation; validation; formal analysis; writing – original draft; writing – review and editing; visualization. **Supriya Prakash:** Methodology; investigation. **Suyog Shaha:** Methodology; investigation. **A. Da Silva‐Candal:** Methodology; investigation; data curation; formal analysis. **Kyung Soo Park:** Methodology; investigation. **Samir Mitragotri:** Conceptualization; funding acquisition; writing – review and editing; supervision; resources; project administration.

## CONFLICT OF INTEREST STATEMENT

N.K., S.P., and S.M. are inventors of patent applications related to the technology described in the manuscript (owned and managed by Harvard University). S.M. is a shareholder and board member of Hitch Bio.

## Supporting information


**Figure S1.** Anti‐tumor effect of NE/CAMPs on subcutaneously implanted GL261.
**Figure S2.** Effect of NE/CAMP treatment on GL261 cell growth.
**Figure S3.** Growth curves of subcutaneous GL261 after each treatment.
**Figure S4.** Efficient suppression of rechallenged subcutaneous tumor by NE/CAMP+aPD‐1.
**Figure S5.** Effect of NE/CAMP+aPD‐1 treatment on the survival rate of orthotopic GL261 mice.
**Figure S6.** Biodistribution of intravenously injected NEs and NE/CAMPs in orthotopic GBM model.
**Figure S7.** The proportion of NEs in blood, draining CLNs, and spleen 4 days after the treatment with NE/CAMPs and aPD‐1.
**Figure S8.** Immunohistological analyses on macrophage phenotypes, FOXP3 or CD8‐positive T cells in GBM tissue.
**Figure S9.** Example gating scheme for the lymphoid arm of immunophenotyping studies.
**Figure S10.** Example gating scheme for the myeloid arm of immunophenotyping studies.

## Data Availability

The data that supports the findings of this study are available in the supplementary material of this article.
