# Supplementary material for: Immunotherapy against glioblastoma using backpack‐activated neutrophils
Source: Bioeng Transl Med. 2024 Aug 13;10(1):e10712. doi: 10.1002/btm2.10712 (PMC11711226; doi:10.1002/btm2.10712)
Supplement: Supplementary file 1 — Figure S1. Anti‐tumor effect of NE/CAMPs on subcutaneously implanted GL261. Figure S2. Effect of NE/CAMP treatment on GL261 cell growth. Figure S3. Growth curves of subcutaneous GL261 after each treatment. Figure S4. Efficient suppression of rechallenged subcutaneous tumor by NE/CAMP+aPD‐1. Figure S5. Effect of NE/CAMP+aPD‐1 treatment on the survival rate of orthotopic GL261 mice. Figure S6. Biodistribution of intravenously injected NEs and NE/CAMPs in orthotopic GBM model. Figure S7. The proportion of NEs in blood, draining CLNs, and spleen 4 days after the treatment with NE/CAMPs and aPD‐1. Figure S8. Immunohistological analyses on macrophage phenotypes, FOXP3 or CD8‐positive T cells in GBM tissue. Figure S9. Example gating scheme for the lymphoid arm of immunophenotyping studies. Figure S10. Example gating scheme for the myeloid arm of immunophenotyping studies. [file BTM2-10-e10712-s001.pdf]

Supplementary information for

Immunotherapy against Glioblastoma using Backpack-activated Neutrophils

Tatsuya Fukuta[1,2,3,#], Ninad Kumbhojkar[1,2,#], Supriya Prakash[1,2], Suyog Shaha[1,2], A Da Silva-Candal[1,2,4], Kyung Soo Park[1,2], Samir Mitragotri[1,2,*]

[1]*Harvard John A. Paulson School of Engineering and Applied Sciences, Harvard University, Allston, Massachusetts 02134, United States*

[2]*Wyss Institute for Biologically Inspired Engineering, Boston, Massachusetts 02115, United States*

Present address: [3]*Department of Physical Pharmaceutics, School of Pharmaceutical Sciences, Wakayama Medical University, 25-1 Shichiban-cho, Wakayama 640-8156, Japan*

[4]*Neurovascular Diseases Laboratory, Neurology Service, University Hospital Complex of A Coruña, Biomedical Research Institute, A Coruña, Spain*

[*]Corresponding author: Samir Mitragotri, Ph.D.

Email: mitragotri@seas.harvard.edu

Harvard John A. Paulson School of Engineering and Applied Sciences, Harvard University

[#]These authors contributed equally to this work

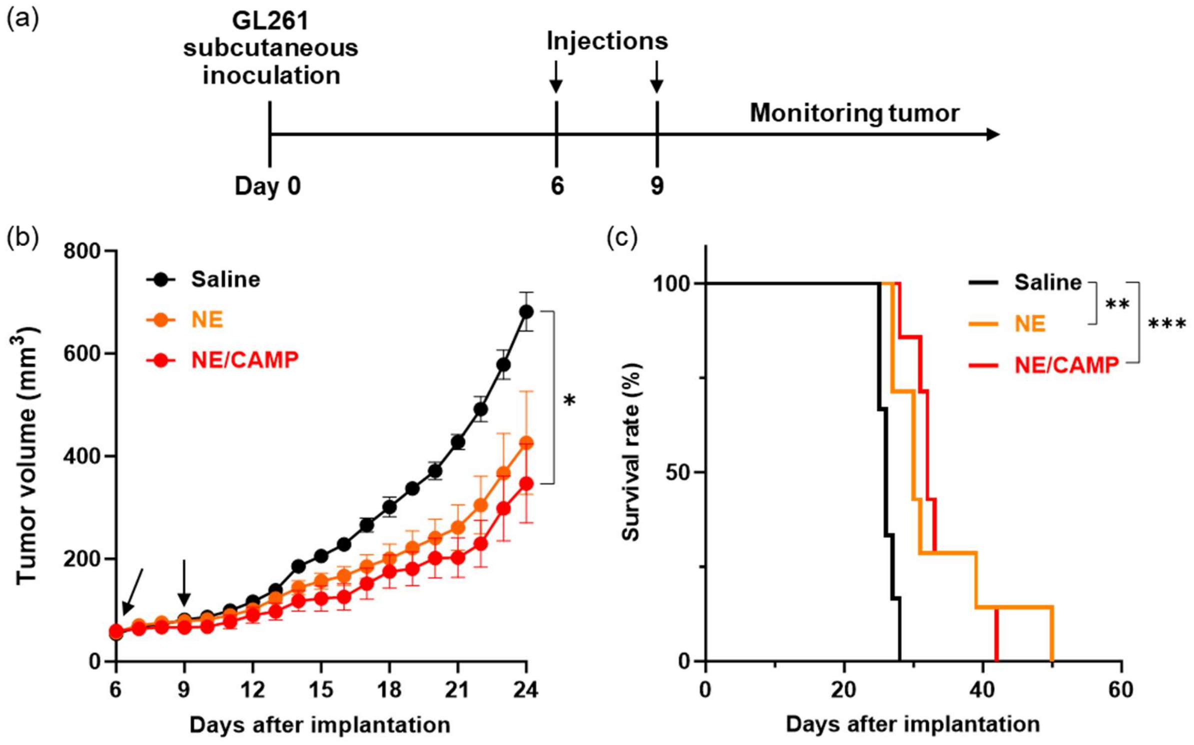

Figure S1. Anti-tumor effect of NE/CAMPs on subcutaneously implanted GL261.

(a) C57BL/6 mice bearing subcutaneous GL261 tumors were intravenously administered NE/CAMPs (2×10^6 NEs/injection), NEs (2×10^6 NEs/injection), or saline two times 3 days apart after average tumor volume reached 50 mm^3, that is days 6 and 9 following tumor inoculation. (b) Tumor volume and (c) survival rate were monitored daily. Black arrows in (b) indicate the days of each treatment administration. Data are the mean ± SEM (n=6 for saline, n=7 for NE and NE/CAMP). Statistical differences among each group in (b) were analyzed by one-way ANOVA with Tukey's multiple comparisons test, and those in (c) were determined by log-rank (Mantle-Cox) test. *P<0.05, **P<0.01, and ***P<0.001 *vs.* saline group.

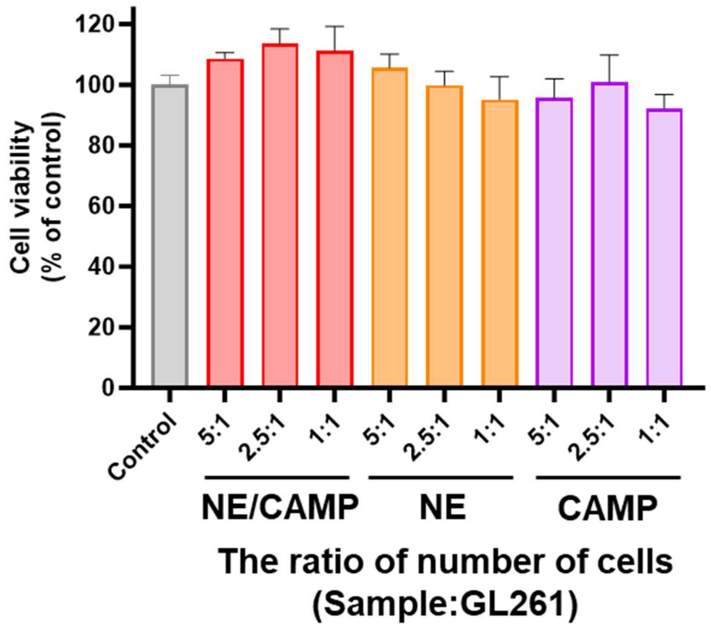

Figure S2. Effect of NE/CAMP treatment on GL261 cell growth

GL261 cells (2×10^4 cells/well onto 96-well plate) were treated with NE/CAMPs, NE, or CAMPs for 24 h. Tumor cells were treated with NEs at a ratio of 1:5, 1:2.5 and 1:1. At 24 h after the sample addition, the media containing each sample was changed to fresh media, followed by additional culture for 24 h. The viable cells were quantified by a WST-8 assay. Data are mean ± SD (n=4).

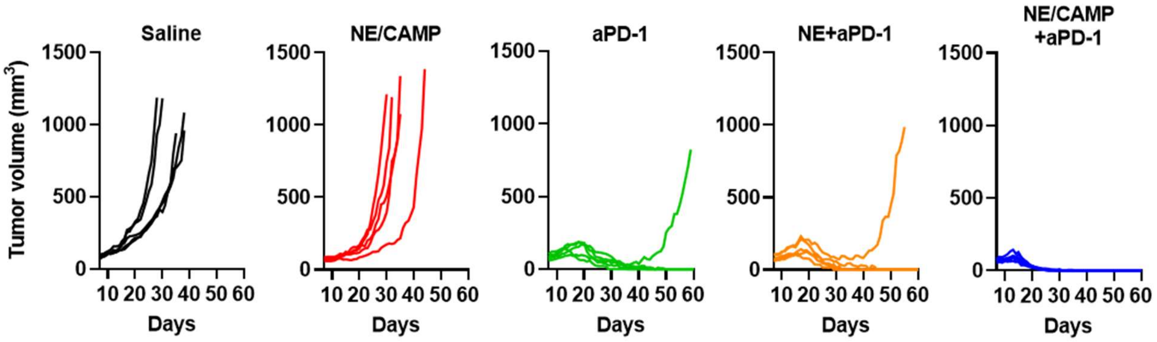

Figure S3. Growth curves of subcutaneous GL261 after each treatment.

Individual tumor growth curves for mice bearing subcutaneous GL261 tumors treated with aPD-1 and/or NE/CAMP.

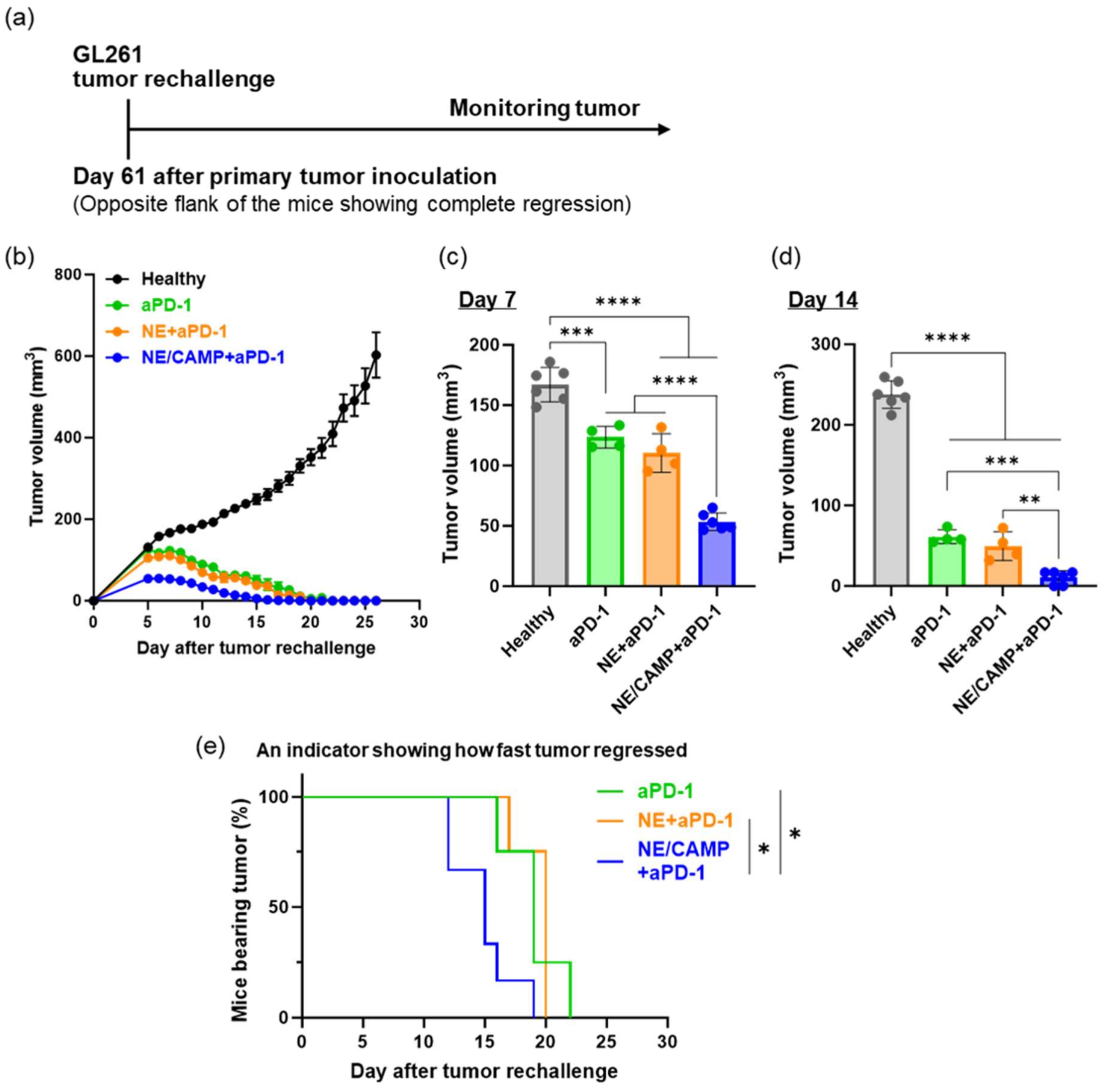

Figure S4. Efficient suppression of rechallenged subcutaneous tumor by NE/CAMP+aPD-1.

(a) GL261 cells (5×10^6 cells/mouse) were subcutaneously implanted into the opposite flank of the mice that showed complete regression of the primary tumors. The same number of the GL261 cells were also subcutaneously injected into healthy mice. (b) Tumor growth curve of all groups. Data are the mean ± SEM (n=6 for Healthy and NE/CAMP+aPD-1, and n=4 for aPD-1 and NE+aPD-1). (c, d) Tumor volume of Day 7 (c) and Day 14 (d). Data are the mean ± SD. Statistical differences were analyzed by one-way ANOVA with Tukey's multiple comparison tests. **P<0.01, ***P<0.001, and ****P<0.0001. (e) The data of Mice with complete tumor regression (%) analyzed with log-rank (Mantle-Cox) test. *P<0.05.

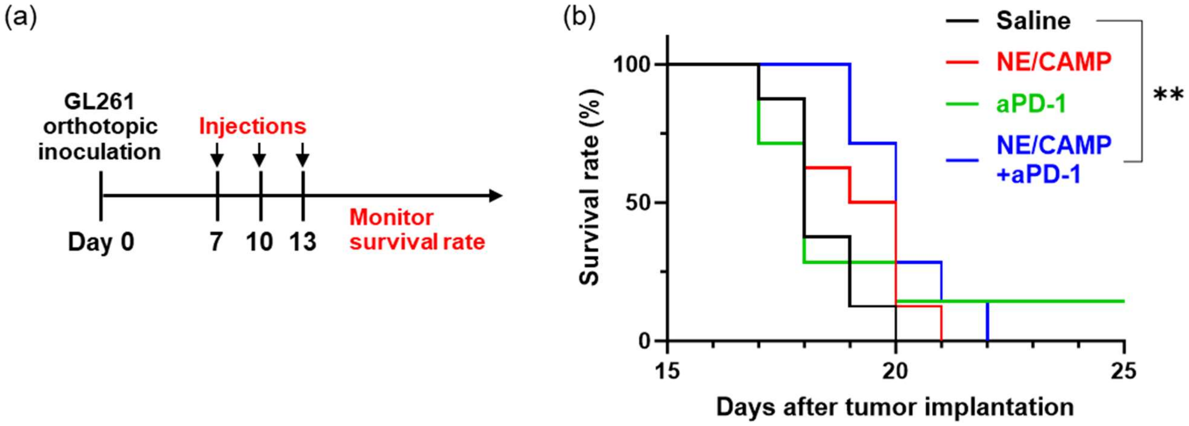

Figure S5. Effect of NE/CAMP+aPD-1 treatment on the survival rate of orthotopic GL261 mice.

(a) Dosing regimen for survival rate study. The mice were treated with NE/CAMPs (3×10^6 NEs/mouse/injection, *i.v.*) and aPD-1 (100 µg/mouse/injection, *i.p.*) on days 7, 10, and 13, followed by monitoring survival rate. (b) Survival rates of mice under different treatments as presented by Kaplan-Meier curves. The mice were monitored daily to measure body weight and to check behavioral conditions after tumor induction. When the mice lost over 20% of body weight and had neurological symptoms such as extreme lethargy and hunched body posture, the mice were euthanized by carbon dioxide inhalation. Statistical analysis was conducted using by log-rank (Mantle-Cox) test. $^{**}P<0.01$.

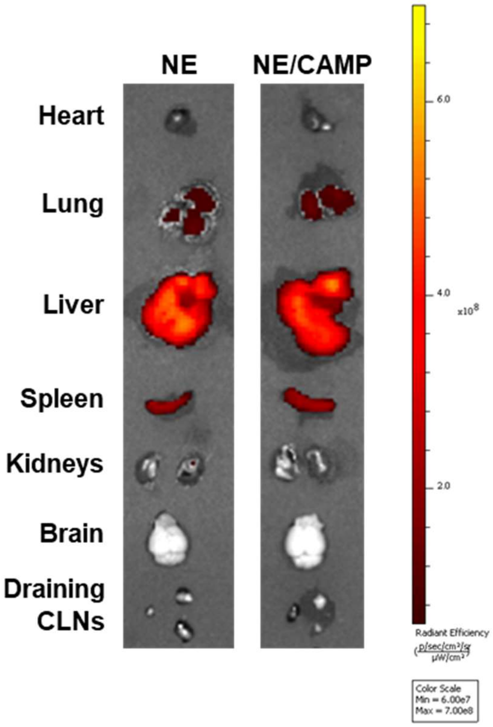

Figure S6. Biodistribution of intravenously injected NEs and NE/CAMPs in orthotopic GBM model.

VivoTrack680-labeled NEs or NE/CAMPs (2×10^6 NEs/mouse) were intravenously administered into the mice bearing orthotopic GL261 at 10 days after tumor implantation. The fluorescence in each organ was determined with the *in vivo* imaging system. The representative images of each group are shown.

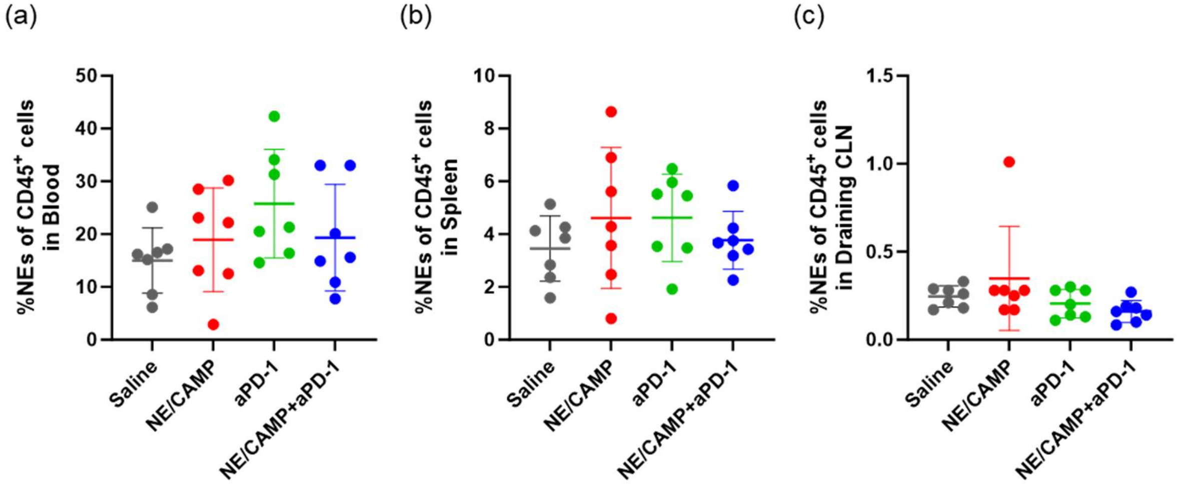

Figure S7. The proportion of NEs in blood, draining CLNs, and spleen 4 days after the treatment with NE/CAMPs and aPD-1.

The mice were treated with NE/CAMPs, aPD-1, saline 7 and 10 days after tumor implantation. At 4 days after injection, NE proportion (%Ly6G^{+} Ly6C^{+} of CD45^{+}) in blood (a), spleen (b), and draining CLNs (c) was analyzed after processing into single-cell suspensions by flow cytometry. The information of the antibodies against Ly6G and Ly6C are shown as below.

Anti-Ly6G antibody; Fluorophore: BV711, Clone: 1A8, Supplier: Biolegend

Anti-Ly6C antibody; Fluorophore: APC-Fire750, Clone: HK1.4, Supplier: Biolegend

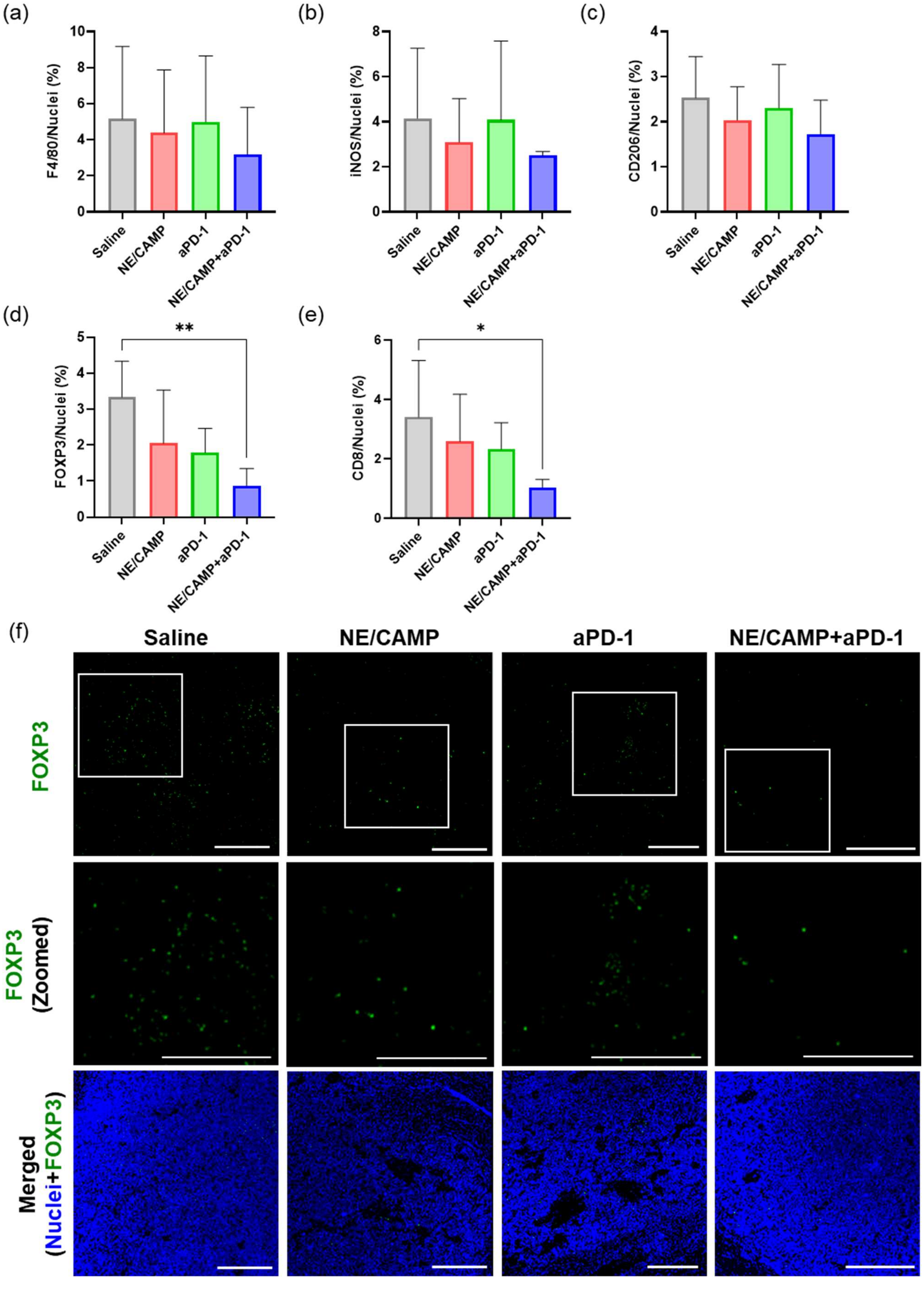
(a)
F4/80/Nuclei (%)
(b)
iNOS/Nuclei (%)
(c)
CD206/Nuclei (%)
(d)
FOXP3/Nuclei (%)
**
(e)
CD8/Nuclei (%)
*
Saline
NE/CAMP
aPD-1
NE/CAMP+aPD-1
(f)
Saline
NE/CAMP
aPD-1
NE/CAMP+aPD-1
FOXP3
FOXP3 (Zoomed)
Merged (Nuclei+FOXP3)

Figure S8. Immunohistological analyses on macrophage phenotypes, FOXP3 or CD8-positive T cells in GBM tissue.

The mice were treated with NE/CAMPs, aPD-1, saline 7 and 10 days after tumor implantation The brains of the mice of each group were also collected on day 14, followed by the preparation of 10-µm frozen brain sections with a cryostat. The frozen brain sections were immunostained for F4/80 (macrophage marker), iNOS (M1 marker), CD206 (M2 marker), FOXP3 (Treg marker), and CD8 with Alexa488-conjugated antibodies. After staining nuclei with DAPI, the fluorescence in the brain sections was observed with Zeiss AxioScan. The quantitative data of the proportion of each marker positive cells to the number of nuclei were analyzed with ZEN 3.3 blue edition software (a: F4/80/nuclei (%), b: iNOS/nuclei (%), c: CD206/nuclei (%), d: FOXP3/nuclei (%)), e: CD8/nuclei (%). Data are the mean ± SD (n=5-7). (e) The representative images of the brain sections stained for FOXP3. Scale bars: 500 µm. The images in the middle row show zoomed images of the area encompassing the white squares in the images in the top row. Following antibodies were used for staining:

Anti-F4/80 antibody; Clone: BM8, Supplier: Biolegend

Anti-iNOS antibody; Clone: CXNFT, Supplier: Biolegend

Anti-CD206 antibody; Clone: C068C2, Supplier: Biolegend

Anti-FOXP3 antibody; Clone: MF-14, Supplier: Biolegend

Anti-CD8a antibody; Cline 53-6.7, Supplier: Biolegend

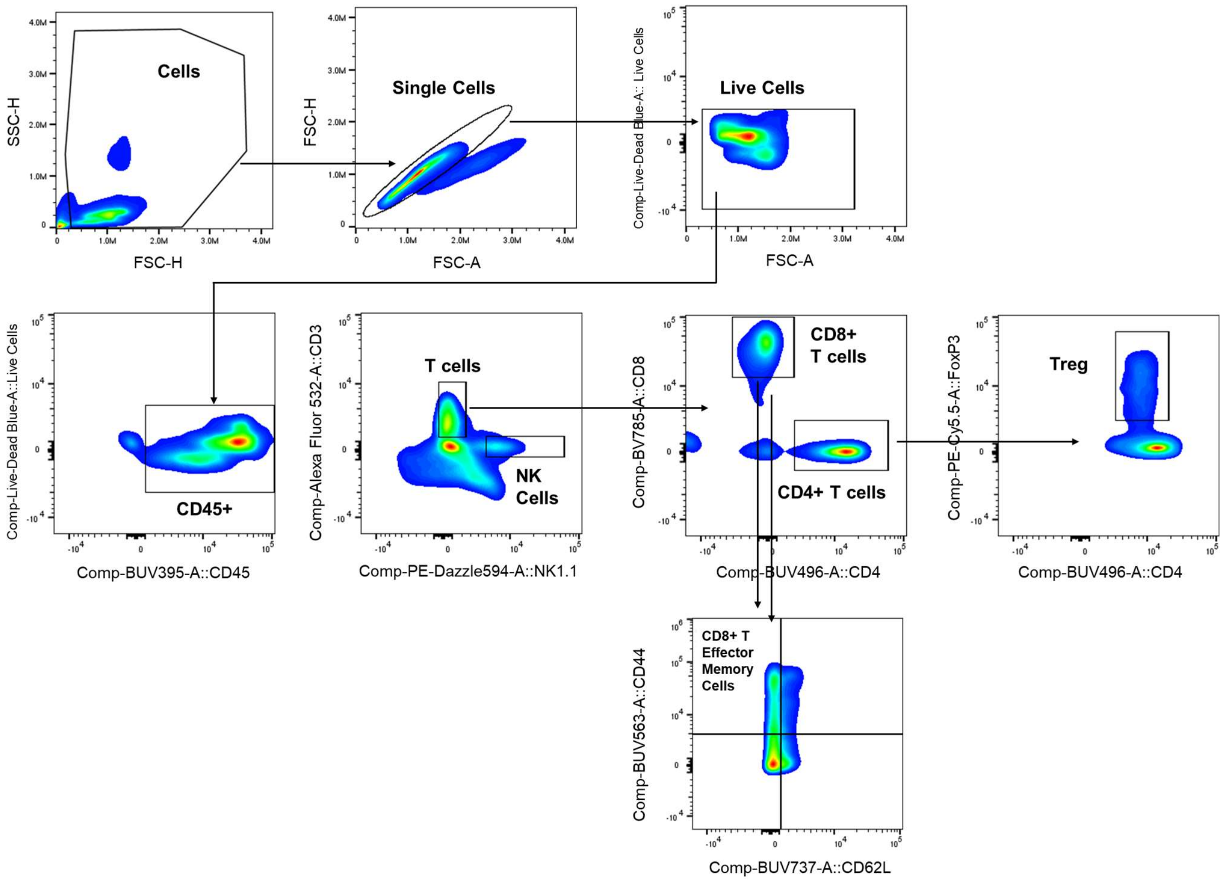

Figure S9. Example gating scheme for the lymphoid arm of immunophenotyping studies.

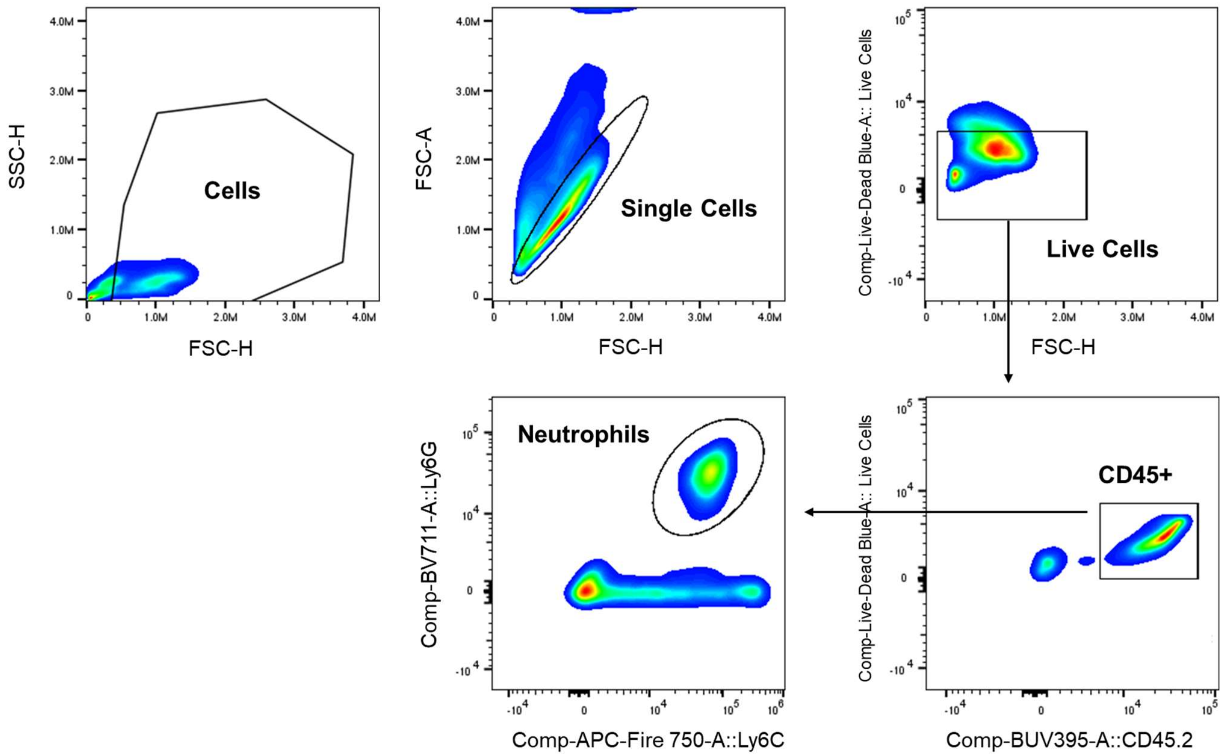

Figure S10. Example gating scheme for the myeloid arm of immunophenotyping studies.
